# The Reasonable Ineffectiveness of Mathematics in the Biological Sciences

**DOI:** 10.3390/e27030280

**Published:** 2025-03-07

**Authors:** Seymour Garte, Perry Marshall, Stuart Kauffman

**Affiliations:** 1 Department Pharmacology and Toxicology, School of Pharmacy, Rutgers University, Piscataway, NJ 08854, USA; 2 Evolution 2.0, Oak Park, IL 60301, USA; evolution@evo2.org; 3 The Institute for Systems Biology, Seattle, WA 98109-5263, USA; stukauffman@gmail.com

**Keywords:** mathematical laws, set theory, third transition

## Abstract

The known laws of nature in the physical sciences are well expressed in the language of mathematics, a fact that caused Eugene Wigner to wonder at the “unreasonable effectiveness” of mathematical concepts to explain physical phenomena. The biological sciences, in contrast, have resisted the formulation of precise mathematical laws that model the complexity of the living world. The limits of mathematics in biology are discussed as stemming from the impossibility of constructing a deterministic “Laplacian” model and the failure of set theory to capture the creative nature of evolutionary processes in the biosphere. Indeed, biology transcends the limits of computation. This leads to a necessity of finding new formalisms to describe biological reality, with or without strictly mathematical approaches. In the former case, mathematical expressions that do not demand numerical equivalence (equations) provide useful information without exact predictions. Examples of approximations without equal signs are given. The ineffectiveness of mathematics in biology is an invitation to expand the limits of science and to see that the creativity of nature transcends mathematical formalism.

## 1. Introduction: Mathematics in Natural Science

This paper takes its title and approach from Eugene Wigner’s classic paper “The Unreasonable Effectiveness of Mathematics In the Natural Sciences” [1], a seminal text on the relationship between mathematics and physics.

The laws of nature are regularities discovered despite the complexity of the world. These laws are surprising due to their invariance, independence from many conditions, and simplicity. They are conditional statements that predict future events based on the present state of the world. The laws are formulated in the language of mathematics, as exemplified by Newton’s law of gravity and Einstein’s laws of relativity [2,3].

Quantum mechanics brought a major transition in science but still follows the Newtonian paradigm: (i) identify the relevant variables; (ii) write equations of motion among those variables in differential form; (iii) define the boundary conditions that specify the phase space of all possible combinations of the relevant variables; (iv) specify the initial conditions; and (v) integrate the equations of motion to deduce system behavior in its predefined phase space.

Newton’s law of gravitation, initially based on limited observations, has proven to be accurate to a remarkable degree. Quantum mechanics, originating from matrix calculations, has been successfully applied to complex problems beyond its initial scope [4,5]. Quantum electrodynamics, a purely mathematical theory, agrees with experimental results with an extraordinary precision [6].

The appropriateness and accuracy of the mathematical formulation of the laws of physics can be considered an empirical law of epistemology. It entices us with the possibility of a single, consistent model encompassing all laws of natural reality. However, it is uncertain whether all physical laws will eventually fuse into a single consistent unit or if there will always be some laws “hanging in mid-air”. The example of the incompatibility between quantum theory and the theory of relativity illustrates the possibility of conflicting theories [7].

## 2. The Paucity of Biological Laws

Despite significant advances in many fields of biological science like systems biology and bioinformatics, biology still lacks the kind of rigorous mathematical models we find in physics and chemistry. The fact that biology is not rich in theory is well known [8]. Of the theories that do exist (such as Darwin’s theory of evolution), most have never been formulated into mathematical laws; they are inexact generalizations. Physics envy is a well-known propensity of many biologists who desire to be working on a more general understanding of whole fields (like cellular biophysics or regulation of gene expression) rather than on exploring empirical details of particular subsystems.

Biology rarely conforms to strict mathematical rules, as there are almost always exceptions to any proposed biological law. There are countless examples of this. Even the idea of a species turns out to be very fuzzy. One definition of a species is a group of animals that can produce viable offspring only by mating with each other. The problem is that there are too many exceptions to this rule to make it mathematically precise, not to mention the majority of living species that do not actually mate. Sharing the same DNA sequence does not work either because individuals within a species do not share exact copies of a complete sequence, and it is impossible to draw the line at a particular degree of sequence variation between different genomes that amounts to a new species.

It is equally difficult to come up with a clear, precise definition of a gene. While early geneticists conceived of genes as discrete units of heredity, we now know that genes are fuzzy entities with indistinct boundaries. Genes overlap, share regulatory elements, are spliced in multiple ways, and function in complex regulatory networks. An allele is a “small” deviation in a genetic sequence in an individual from the normal average in the population. But, how many base–pair sequence changes are required to cross the boundary between a new allele and a new gene? This is completely variable and depends on the location of the mutations making an allele, the function of the gene and its various segments, and other factors. No mathematical equation can define the maximum number of sequence changes before we obtain a new gene.

We acknowledge that it is not the fault of mathematics that the definition of the word “gene” is sloppy. That said, many other biological concepts resist clear definitions amenable to mathematical treatment. These include being alive and being an individual, as well as agency, inheritance, intelligence, sentience, and cognition.

For some areas of biological science, great mathematical minds have, for quite some time, tried to apply their skill to producing a kind of law that is, as Wigner [1] put it, “true everywhere on the Earth, was always true, and will always be true.” There have been a few successes and many failures [9,10].

Evolutionary biology is a prime example. We certainly have a powerful model, one that has been formulated, improved upon, and expanded over the years to include data and concepts from many disciplines and has been empirically demonstrated in the lab [11,12,13,14,15]. And yet, where is the law of evolution? Why can we not formulate a mathematical treatment of the evolutionary process, including the inheritance of genotype, selection of the fittest phenotype, and fixation of that genotype in the population?

Of course, we have equations that can approximate some of the steps fairly well but not the whole process. One of the key concepts in evolutionary theory is fitness, which underlies the principle of natural selection. The problem is that fitness has no precise scientific definition in biology. It is impossible to predict a priori what phenotypic trait will render a particular individual or population living in a particular environment more or less “fit.” The only way we can quantify fitness in any population is by the comparative measurement of survival rates and reproductive success. This means that any mathematical description of the mechanism of evolution by natural selection is ultimately circular: a tautology.

## 3. Reasons for the Ineffectiveness of Mathematics in Biology

The point of making a mathematical theory is to tame what seems to be a wild, complex mess of unconnected data into a tidy, manageable package. But what if tidy, manageable packages are something alien to biology? The problem may be that biology, unlike physics, is simply not amenable to mathematical description.

Of course, in practice, mathematics does not always describe the real physical world very well either. It is well known that predicting weather more than a few weeks in advance is impossible, as shown by chaos theory [16]. What mathematics does is describe models of the real world perfectly, and the best laws for the models are used for real-world calculations. If the model is very good, the laws also work well in the real world.

Perhaps this is the problem. Perhaps perfect bio-mathematical models are impossible. In evolution, for example, one can develop a model, but as soon as we have one that can be predicted by an equation, we run into major difficulties.

## 4. Biology Beyond Mathematics

Kauffman and Roli [17], Marshall [18], and Louie [19] have issued mathematical proofs that a “Laplacian” model of biology, named for Laplace who theorized [20] that the future can be determined by perfect knowledge of the past, is impossible. While few scientists insist that the universe is 100% deterministic, the manner in which mathematics is treated in science still implies that the Laplacian model is true. A good definition of mathematics is a system of logical deductions from a set of premises (axioms) and rules to arrive at them. However, as we discuss below, the reality of biological science renders the normal axioms and rules of standard mathematics inapplicable, just as they would be for numerous examples of phenomena that are outside the province of computability.

We need to make clear from the outset that our central assertions are not grounded in scientific experiments which are subject to argument, interpretation, and counterexamples. They are mathematical proofs and thus logically and necessarily true.

Our arguments are closely related to Godel’s incompleteness theorem and Turing’s Halting Problem [21,22,23]. In other words, we are not saying “Biology so far has resisted rigorous mathematical formalization, therefore it must be impossible and it is time to give up”. Rather, we are saying the following: because mathematics itself is incomplete [24], any scientific description invoking mathematics is also necessarily incomplete. This is well known in the field of mathematics [25,26]. We will develop this argument further below.

Biology exercises agency and cognition and generates negative information entropy [27]. Organisms demonstrably exercise choice and harness affordances in ways that non-living systems do not. While not absolutely provable in the mathematical sense, in this paper, we offer both supporting evidence and criteria for how to scientifically disprove our hypothesis.

## 5. Computationally Intractable Problems vs. The Hard Problem of Biology

There are two levels of challenge here which we need to clearly distinguish. The first level of challenge is the “Computationally Intractable” problem: even the simplest problem of three interacting bodies cannot be solved rigorously mathematically. But, this state of affairs alone is not a reason to declare the inefficiency of mathematics. Any more or less complex processes in physics (the behavior of bulk solids, the turbulent motion of liquid, the occurrence and shape of a crack in a solid, multi-electron atoms, the description of high-temperature superconductivity, etc.) also demonstrate “the striking inefficiency of mathematical models”.

“Computationally intractable” or “NP-hard” problems in computational complexity theory are solvable in principle but require computational resources that grow exponentially with the size of the input, making them effectively impossible to solve for any but the smallest cases. A classic example is the “traveling salesman problem”: finding the shortest possible route through a set of cities.

Many times, this is a limitation of existing mathematical formalisms: problems where an analytic solution is not available but a numerical solution can still be found given enough data points and computing power. Many problems in physics can in fact be solved in a Laplacian fashion, at least in principle. But, in practice, predicting the weather 6 months in advance is, for all practical purposes, impossible, so we use heuristics instead.

The difficulty is not because mathematics is not applicable but sometimes because scientists have only just begun to study complex systems (this science is not yet 100 years old). We agree that when the principles are understood, and sufficient data are supplied, the corresponding mathematical result will appear.

Take, for instance, protein folding (which, arguably, lies at the interface of physics and biology). This problem has now been nearly completely solved using computational methods. However, this has not been performed by taming the mess of crystallographic data into a tidy package: anything but! The algorithms are not transparent, but the process is at least approximately computable. The fact that we have developed useful algorithms proves that.

The fact that “tidy, manageable packages are alien to biology” does not all by itself mean that biology is not amenable to mathematical description. At this point, we might name our paper “The Reasonable Ineffectiveness of Present Science in Complex and Nonlinear Phenomena”.

But, in biology, we encounter a second level of difficulty. The problem is that, as James Shapiro says, “All Living Cells are Cognitive” [28,29,30,31]. This is, by definition, a mathematically intractable problem. It is equivalent to Chalmers’ Hard Problem of consciousness [32]; Walker and Davies call it the Hard Problem of Life [33,34].

Computationally intractable problems still follow deterministic rules: we can write the algorithm, we just cannot run it in reasonable time. The Hard Problem of biology involves genuine choice and agency that cannot be reduced to an algorithm at all.

This maps well to the distinction between weather prediction (computationally intractable but theoretically deterministic) and biological agency (fundamentally non-algorithmic).

We believe that it is more sensible to say that mathematics determined the big bang and formation of the earth than to say those things created mathematics. This is because pure physics and chemistry do always seem to obey mathematical laws. This is the point of Wigner’s paper. A great many mathematicians believe mathematical laws precede material objects. As Leopold Kronecker famously said, “God created the integers, all else is the work of man” [35].

Biology, on the other hand, performs induction which by definition creates mathematics. Organisms make inferences where exact answers cannot be precisely known [12,36,37]. This means biology creates in ways that nonliving matter does not.

The thesis of this paper is that biological organisms really do create mathematics and, in effect, choose axioms, both implicitly and explicitly. Humans explicitly chose a base-10 number system. Many other numbering systems are possible.

The exercise of agency does not just apply to humans. Levin [12] reports that in newts, the construction of kidney tubules demonstrates remarkable adaptability depending on the ploidy (chromosome number) of the animal, which affects cell size.

In normal newts, kidney tubules are typically formed by the interaction of 8 to 10 small cells in a cross-section. These cells communicate and coordinate with each other to create a tubule with a lumen of a specific size. This process relies on cell-to-cell communication mechanisms.

When newts are artificially made polyploid, their cell size increases significantly due to the higher chromosome number. Despite this increase in cell size, the kidney tubules still maintain the correct lumen diameter. As the cells become larger, fewer cells are required to form the tubule. For instance, instead of 8 to 10 cells, a smaller number of larger cells are used to achieve the same geometric structure.

In cases where the cells are made extremely large, the system adapts even further. Instead of relying on cell-to-cell communication, a single large cell wraps itself into a “C” shape to form the tubule. This process uses a completely different molecular mechanism—cytoskeletal bending—rather than the usual cell-to-cell constructions.

This highlights the plasticity and adaptability of the biological system, as it can creatively deploy different mechanisms to achieve the same structural goal (a kidney tubule with the correct lumen diameter) despite drastic changes in cell size.

This is an affordance, an example of a biological system choosing an alternate set of axioms and using them to compute a new result in the face of a threat. We use the term “compute” here deliberately: yes, the organism is computing, but this comes after a choice has been made to take advantage of an affordance.

Similarly, when planarian flatworms are exposed to barium, a non-specific blocker of potassium channels, their heads undergo a process of degeneration due to the inability of neural tissues to maintain normal ionic balance. This results in the heads not developing or functioning correctly, and in some cases, the heads even “explode” or degrade entirely.

When these planarians are kept in a barium solution, their remaining tails regenerate new heads that are completely resistant to barium. This occurs immediately. This adaptation is particularly fascinating because barium is not a substance that planarians encounter in the wild, meaning that there has been no evolutionary pressure to develop a specific response to it. This is a novel response to an unforeseeable event. Marshall [18] calls this an “undecidable proposition” after Gödel and Turing [22].

The transcriptomic analysis of these barium-adapted heads reveals that only a small number of genes show altered expression compared to wild-type heads. This indicates that the cells in the planarian system are capable of navigating transcriptional space to identify and activate a minimal set of genes necessary to resolve this novel physiological stressor. The new heads are fully functional and barium-insensitive, showcasing the extraordinary regenerative and adaptive capabilities of planarian cells [38].

Kauffman and Roli [17] prove that it is not possible to apply set theory to affordances, which are features of the world that can be used to perform something useful [39]. For example, it is not possible to list all the possible uses of an engine block. If we cannot list all the uses of X or of Y, the first axiom of set theory fails: two sets are identical if and only if they have the same members. But, if we cannot list all the uses of X or of Y, we cannot prove that their uses are identical. The number of uses of an engine block is indefinite. You can use it to make an engine but also to crack open a pineapple or for innumerable other purposes. It is not possible to order the “uses of X”. Those uses are merely a nominal scale. Further, it is not possible to deduce one use of X from another use of X. These three properties define indefinite. A plurality of items is indefinite if and only if i. the items cannot be listed; ii. the items cannot be ordered relative to one another; and iii. the items cannot be deduced from one another.

“Uses of X”, that is, affordances, are indefinite. Indefinite does not map to the real line. The evolving biosphere plucks novel emergence from the indefinite.

Walker [40] dismisses this problem, saying that “We do not need to iterate over all the functions a screwdriver or any other object might have, because the vast majority of those are irrelevant to the future of our biosphere”. She is right that most are irrelevant, but this is indeed the problem. A “Laplacian” predictive mathematical model necessarily predicts every future step from the present. In the next paragraph, she acknowledges that function is a notoriously tricky problem to define. This is why mathematics cannot predict it. The problem in principle cannot be solved within mathematics for the same reason that mathematics cannot generate itself.

Just as all mathematical formalisms require axioms which must be chosen by an agent, any attempt to enumerate a list of affordances of an engine block (“list all objects you can break with an engine block in alphabetical order”) requires an agent to identify all possible objects, choose an alphabet, name the objects, and order them according to an arbitrary set of rules. Even an activity as “simple” as counting requires agency. Counting apples requires you to decide what is and is not an apple. Mathematically, this is an undecidable proposition.

What we can demonstrate empirically but cannot absolutely prove (thus making this aspect of our argument science and not mathematics) is our insistence that biological agents are indeed making choices, that humans really are choosing which scientific theories are good or bad, and that our dogs really are choosing whether to urinate in the living room or back yard. “There is nothing so amusing as a guy whose purpose is to convince you that there is no purpose” [41].

We admit we cannot absolutely prove that the universe is not deterministic. Perhaps all choices really are illusions. We can only point out that such a universe would be more predestined than anything imagined by most creationists.

Walker and Davies [33] counter this flavor of determinism by saying that “To explain a world as complex as ours which includes things like bacteria and at least one technological civilization with knowledge of things like the laws of gravitation requires that the universe have a very special initial state indeed. The degree of ‘fine-tuning’ necessary to specify this initial state is unsatisfactory and becomes ever more so the more complex the world becomes. One should hope for a better explanation than simply resorting to special initial conditions (or for that matter stochastic fluctuations, which in many regards are even less explanatory)”. In such a universe, not even a scientist’s most cherished theories would truly be their own.

Marshall [18] uses Turing mathematics to prove (and not merely hypothesize) that predictions about the future, assigning meaning to symbols, inductive reasoning, axioms in mathematics, negentropy, measurement, and perception are all undecidable propositions, equivalent to Turing’s Halting Problem. All require choices that are not computable from prior states; thus, biology transcends the limits of computation [18].

Louie [19], drawing on Rashevsky [42], uses relational biology to demonstrate mathematically that some biological properties emerge from relationships that cannot be reduced to their physical components. Relational descriptions can apply to a large class of functionally identical but physically quite distinct systems.

For instance, in electrical circuits [43] and hydraulic systems [44], the same mathematical relationships govern the flow of entirely different physical quantities: electrons and water molecules, respectively. Similarly, metabolic networks in cells exhibit identical regulatory patterns despite utilizing different specific enzymes and substrates across species. This mathematical abstraction allows us to identify universal organizational principles that transcend physical implementation.

Rashevsky demonstrated this concept through what he called the “principle of biological epimorphism” [45,46]. One of his most famous examples involved comparing the digestive systems of different organisms. He showed mathematically that despite vast differences in physical structure—from the simple gut of a hydra to the complex digestive tract of mammals—these systems could be mapped to the same relational model describing the basic functions of nutrient absorption and waste removal. The physical implementations varied dramatically, but the fundamental relationships between inputs, transformations, and outputs remained invariant. This demonstrated that biological functions could be understood at an abstract relational level, separate from their specific physical manifestation.

While on the surface these mathematical isomorphisms appear to contradict our thesis, they in fact support it. Remarkable mathematical relationships hold given deliberate and careful choices as to what level of the system we choose to study and which attributes we choose to include and ignore in our model.

The golden ratio and fractal geometry are used to describe shape. Differential equations [47] are used to describe population development, growth, etc. Probability theory is widely used in genetics. Game theory is used in models of behavior. Models of intelligence are based on discrete mathematics and algebra. Processing any data in biology uses mathematical statistics. We are not throwing mathematics under the bus.

We are saying that (1) a chosen subset of mathematics always has to be selectively applied to a chosen subset of the physical system in question and (2) that biology generates its own choices and cognitive models as the organism responds to its environment; non-living things do not.

Louie says that “Relational biology is mathematical organization seeking realizations, and reductionistic biology is physicochemical processes seeking models. Relational biology decodes; reductionistic biology encode. The antithesis of relational biology is this presumptuous reductionism (that ‘biology is chemistry,’ or exclusive adherence to the bottom-up causal chain)” [19].

Kauffman and Roli [48] say that “it is not possible to apply set theory to affordances; therefore, we cannot devise a set-based mathematical theory to deduce the diachronic evolution of the biosphere.” They conclude that “Any theory requiring the notion of sets that tries to model the diachronic emergence of affordances is inherently flawed. …(the) evolution of the biosphere, besides the impossibility of being entailed, is inherently not mathematizable… the world is not a theorem. Apologies to Pythagoras, Plato, Neoplatonists, Newton, Bohr” [48].

## 6. Defining Life

The question of what constitutes life is famously contentious, with biologists offering a multitude of conflicting definitions. Even seemingly basic distinctions, like that between a symbiont and a parasite, dissolve under scrutiny. As Denis Noble put it, in biology, “there is no privileged level of causation” [49]; causal relationships exist at all scales in a dizzyingly complex web of interactions. Even viruses are capable of limited sorts of engineering to achieve their own ends [50]. There are no simple linear causes and effects.

Fitness is only one example of the problems facing the theoretical biologist in the construction and use of models. Take the cell, or any functional cellular component like a ribosome or chloroplast. We know a great deal of the detail of the molecular mechanisms of protein synthesis and photosynthesis, and we can make impressive videos and elaborate simulations, but how can such processes be mathematically modeled with any degree of accuracy?

A number of scholars have shown that biology transcends the limits of computation [19,46,51]. These papers show that if biology were computational, it would only be able to perform deductive logic. Induction and inference would, by definition, be impossible. But, living things indisputably sense their environment, make decisions in the face of ambiguity, make predictions, and exercise agency [31,52]. Organisms evolve irreducibly complex, novel structures in real time in response to situations that have never occurred in evolutionary history [53].

Biological adaptations emerge differently than human-engineered designs. They can appear as unexpected, fully integrated systems, such as real-time endosymbiosis and the formation of polyploid giant cancer cells, where an immune cell fuses with a somatic cell and enters the bloodstream, eventually metastasizing and leading to N-stage cancer [54]. The fitness of the entire organism influences which adaptations evolve, through a continuous process of functional emergence and active integration.

Living cells constitute a new class of matter [55,56] and organization of processes that is a new union of thermodynamic work, catalytic closure, and constraint closure. The implications of this cannot be overstated. It means, in principle, that it is not merely difficult but impossible to reduce biology to equations. Biology does not merely obey mathematics; it creates mathematics [17,57].

This further means that biology is fundamentally different from AI and computers. All existing AI systems, no matter how sophisticated, run on traditional Turing-like binary architectures, which means that their computations are, by definition, deductive and not inductive [23]. Thus, AI making an inference is “synthetic induction” which means, by the definition of a Turing machine, that it is deductively calculating a possible answer to the question, perhaps in a Bayesian-like manner, and may incorporate random number generators to select between possibilities. Keep in mind that popular AI models are trained on large portions of the entire internet; so, given that, their ability to compute “synthetic inferences” might not be as impressive as it appears.

Contrast this with a planarian embryo that develops a barium workaround in a few hours, when such an event may have never occurred before in history.

The biosphere is autopoietic. It constructs itself and evolves new possibilities and adaptations that cannot be prestated or deduced. The 21st century in biology forces us beyond the Newtonian paradigm to confront the non-deducible creativity of evolving biospheres. This invites us to see and live in the creative mystery of nature in a new way and may point to a fundamental truth behind the enormous difficulty of reducing biology to mathematics.

## 7. The Adjacent Possible

One compelling argument for a post-algorithmic view of life comes from the fact that biological systems are constantly exploring an ever-expanding space of possibilities, where each innovation opens up new opportunities for further innovation [58]. Evolution is not the deterministic output of a mathematical process but a creative exploration of affordances which are chosen by the organism [48]. The complexity and diversity of life is not the result of a pre-specified algorithm but the outcome of an open-ended, non-algorithmic process of environmental sensing and emergence.

In an ergodic system, all possible states will eventually be explored, given enough time. But, the biosphere is non-ergodic: it can never exhaustively explore all permutations in any relevant timescale. The non-ergodicity of biology is central. The specific trajectory of evolution is radically contingent rather than converging on one optimal mathematical solution. The system does not obey a fixed set of equations but continuously creates new rules and possibilities.

## 8. Potential Solutions to the Dilemma

Our principal argument has been that mathematical laws based on set theory and standard equations allowing for calculations of outcomes based on a set of parameters are very often impossible in biological science. Mutations such as frame shifts, large deletions and insertions, transposon insertions, and even some point mutations have had unexpected and dramatic effects on protein functions. These include changing the substrates for catalytic activity, altering binding activity and leading to formation of new receptors, and new activities. The ability to predict in advance whether any specific mutation might produce an entirely novel or radically altered phenotype of a protein is far beyond even hypothetical possibility, discussed above, involving the impossibility of computing all possible affordances for structural and functional changes due to mutation [48].

We can label such unknown and unpredictable mutational effects as X, where X_2_, for example, is the new activity of the protein after mutation that affects the phenotype X. As it is unpredictable in nature and degree, the best we can do is try to imagine a range for the value of the fitness of X_2_. The minimum value must be 1, since anything less than 1 would have no effect on evolution. If the maximum value of the range is MX_2_ then the range, which is also the degree of uncertainty in the value of X_2_, is MX_2_−1 = ΔX.

There are an unknown number (*n*) of possible mutational effects denoted as X. So, for the fitness of the total phenotype after *n* mutational effects, we can write a biological analogy to Heisenberg’s uncertainty principle:

$$F_{m}=\prod_{i=1}^{n}{\Delta X}_{i}\geq 1$$

There are some critical differences between this biological uncertainty principle and one of the bedrocks of quantum theory. The most important of these is that Heisenberg derived his equation from known formulas in physics whereas in our case the formula is based on inherent uncertainty in the effects of mutations and is not derivable from previous mathematics. And, of course, it could be written in many different ways.

As it stands, the formula states that the product of all mutational effects on fitness, none of which are knowable, must be greater than the constant, 1 (which is the baseline for relative fitness), in order to persist in a population and lead to evolutionary change. As is the case for the momentum and position of an electron, the value of fitness after a mutation is not computable.

The apparent ineffectiveness of mathematics in biology has led many to argue that living systems are not purely mechanistic and algorithmic. The papers referenced above issue formal mathematical proofs that this is true. There is a rich collection of the recent literature supporting this [46,49,59]. At the very least, our present understanding of the laws of physics is incomplete, and the missing ingredients may not be fixed mathematical “laws” as traditionally conceived.

It might be necessary to either (1) invent a new kind of non-deductive mathematics or formalism conducive to describing biological reality and the unique complexity of living systems (as was performed a number of times for physics) or (2) take a cue from other fields that have developed systematic, testable theories without forcing everything into mathematical models.

Examples of the second approach include the Austrian school of economics [60] that explicitly rejected the mathematical modeling of human economic behavior in favor of “praxeology”—the study of human action and choice—as well as case law, or common law [61], and business strategy consulting [62,63,64,65,66].

Bruce Henderson [67] developed the Growth–Share matrix in the 1960s which ingeniously divided all businesses into four categories: Stars, Cash Cows, Question Marks, and Dogs. He showed that the vast majority of business growth comes from Stars, companies that are leaders in markets growing 10% or more per year. Henderson’s simple heuristic has been used successfully by investors for decades, despite the fact that it is as far from a “Laplacian” model of business as there can be. After all, a corporation, the markets it exists in, and its assets, processes, and employees are vastly more complex than any single biological organism. To exhaustively model any of that would be hopeless. The power of a heuristic is precisely in what it ignores.

Similarly, physiologist Denis Noble says that “If I have your blood pressure, heart rate, height and weight, I know more about your health than an analysis of your entire genome” [68].

This forces us to stretch the boundaries of methodological naturalism. Since biology does not reduce to mathematical formalisms, we need new criteria for identifying causal relationships and judging scientific theories. Much like this happened with mathematics in the aftermath of Gödel; rather than continuing to insist on exhaustively quantifying scientific models, we may need to make peace with irreducible fuzziness, using qualitative and philosophical reasoning alongside quantitative methods. We may have to come to terms with the fact that some of our most cherished models simply do not work.

Choosing which data to pay attention to is far more valuable than collecting large amounts of data.

We have seen the difficulty in biology of using mathematics that requires the two sides of an equation to be numerically equivalent because biology is always in disequilibrium. There are probably no equal signs in biology. Biological mathematics is likely based entirely on inequalities.

However, this does not mean that mathematical analysis is entirely useless in biology. While, for example, the phenotypic effect of a particular DNA mutation may almost never be predictable, this does not mean that symbolic mathematical relationships can never be used for biological laws but means that equations of the form A = B, where A and B possess precise numerical attributes, are seldom absolutely true. For example, many theoretical biology papers include the derivation of equations to account for a host of biological phenomena [13]; it is quite likely that such formulas, when applied to real-world biological systems, prove only approximately valid.

Given the evidence we have presented that Laplacian mathematical thinking is not warranted for building models in biology, it might be more appropriate to consider the use of formulas that avoid equal signs. For example, if A is the growth rate for a cell population, we can say that continued survival of the population requires that

A > 1.

This does not specify what the actual value of A must be. Statements of probability can also be a useful basis for biological formulas. Below is an example:

Prob(Dt) > 0

or the probability of death per unit time is greater than 0, and the integral of Prob D as t approaches infinity = 1, implying the absence of immortality (life is not conserved).

The use of functions without precise definitions of the functions is also often quite common in biological theoretical work, since it suggests that a variable is dependent on one or more particular parameters without defining the precise nature of that dependence.

A = f(B).

For example, the probability that two populations of the same species will evolve into two separate species is a function of the differences in the environments of the two populations and inverse to a function of the degree of interbreeding between the two populations.

These laws are, by their nature, inexact and indeterminate, which reflects the reality of biology as presented above in the biological analogy of the uncertainty principle in physics. Such laws, while precisely non-predictive, do provide useful information that can be empirically confirmed.

Marshall [21,57] argues that causation in biology is cognition -> codes -> chemicals, running in the opposite direction of the standard reductionist model which is chemicals -> codes -> cognition. A single empirical example of a chemical process producing coded information would falsify the thesis of this paper.

The authors of this paper believe that this problem is solvable, not through traditional chemistry models but through what we might call “undiscovered laws of physics” that underly consciousness and cognition.

## 9. Conclusion—A Fork in the Road

Eugene Wigner concluded his original paper [1] by saying the following:

“A much more difficult and confusing situation would arise if we could, someday, establish a theory of the phenomena of consciousness, or of biology, which would be as coherent and convincing as our present theories of the inanimate world… Furthermore, it is quite possible that an abstract argument can be found which shows that there is a conflict between such a theory and the accepted principles of physics. The argument could be of such abstract nature that it might not be possible to resolve the conflict, in favor of one or of the other theory, by an experiment. Such a situation would put a heavy strain on our faith in our theories and on our belief in the reality of the concepts which we form. It would give us a deep sense of frustration in our search for what I called ‘the ultimate truth’”.

His concern was that we might end up with two equally valid but mutually contradictory descriptions of reality—one for the living world and one for the physical world—with no way to resolve which is “more true.”

We have arrived at precisely that point, except we know that the biological perspective is the greater truth. We can say this definitively because without conscious living human agents to propose axioms and hypothesize physical laws, there would be no abstract mathematics for Wigner to discuss in the first place.

The ineffectiveness of mathematics in biology represents a fork in the road in the history of science. We are at the threshold of a “third transition” [17], where the Newtonian clockwork paradigm that was overturned by quantum mechanics is again transformed by the unruly creativity of life. Whereas classical physics shoehorned the world into tidy equations and phase spaces, biology beyond mathematics will have to grapple with fuzziness, context-dependency, and open-ended emergence. This shift will require not just new mathematical tools but a new scientific epistemology that can accommodate life’s creative freedom.

In practical terms, this reevaluation of biological science has major implications for medicine, ecology, and biotechnology. If the dream of reducing life to precise mathematical models is misguided, then we must cultivate a more holistic and less reductive approach in these domains.

Ultimately, dethroning mathematics as the infallible language of biology allows us to step back in humility and wonder. By recognizing the ineffable creativity of life, we move closer to appreciating biological systems as they are, not as we want them to be for the sake of conceptual convenience.

This shift in perspective does not diminish the achievements of mathematical biology. Rather, it places them in a wider context, reminding us that nature’s creativity is vast, transcending any mere formalism. The reasonable ineffectiveness of mathematics in biology, then, is not a failure but an invitation: an invitation to expand our scientific worldview, embrace the unknown, and learn anew to be astonished by the fecund creativity of the living world.

## Data Availability

This article does not contain any additional data.
